# Co‐occurring orchid species associated with different low‐abundance mycorrhizal fungi from the soil in a high‐diversity conservation area in Denmark

**DOI:** 10.1002/ece3.10863

**Published:** 2024-01-31

**Authors:** Ida Hartvig, Chatchai Kosawang, Hanne Rasmussen, Erik Dahl Kjær, Lene Rostgaard Nielsen

**Affiliations:** ^1^ Forest and Landscape Ecology, Department of Geosciences and Natural Resource Management University of Copenhagen Copenhagen Denmark; ^2^ Center for Evolutionary Hologenomics, Globe Institute University of Copenhagen Copenhagen Denmark; ^3^ Smithsonian Environmental Research Center Smithsonian Institute Edgewater Maryland USA

**Keywords:** Ceratobasidiaceae, ITS metabarcoding, orchid mycorrhizal fungi, plant hologenomics, soil mycobiome, Tulasnellaceae

## Abstract

Plant–fungal interactions are ubiquitous across ecosystems and contribute significantly to plant ecology and evolution. All orchids form obligate symbiotic relationships with specific fungi for germination and early growth, and the distribution of terrestrial orchid species has been linked to occurrence and abundance of specific orchid mycorrhizal fungi (OMF) in the soil. The availability of OMF can therefore be a habitat requirement that is relevant to consider when establishing management and conservation strategies for threatened orchid species, but knowledge on the spatial distribution of OMF in soil is limited. We here studied the mycorrhizal associations of three terrestrial orchid species (*Anacamptis pyramidalis*, *Orchis purpurea* and *Platanthera chlorantha*) found in a local orchid diversity hotspot in eastern Denmark, and investigated the abundance of the identified mycorrhizal fungi in the surrounding soil. We applied ITS metabarcoding to samples of orchid roots, rhizosphere soil and bulk soil collected at three localities, supplemented with standard barcoding of root samples with OMF specific primers, and detected 22 Operational Taxonomic Units (OTUs) putatively identified as OMF. The three orchid species displayed different patterns of OMF associations, supporting the theory that association with specific fungi constitutes part of an orchid's ecological niche allowing co‐occurrence of many species in orchid‐rich habitats. The identified mycorrhizal partners in the basidiomycete families Tulasnellaceae and Ceratobasidiaceae (Cantharallales) were detected in low abundance in rhizosphere soil, and appeared almost absent from bulk soil at the localities. This finding highlights our limited knowledge of the ecology and trophic mode of OMF outside orchid tissues, as well as challenges in the detection of specific OMF with standard methods. Potential implications for management and conservation strategies are discussed.

## Graphical abstract

All orchids establish obligatory symbiotic relationships with specific fungi for germination and early growth, contributing to ecological niche differentiation. This investigation revealed that three co‐occurring terrestrial orchid species engaged with distinct fungal partners. The identified fungi were detected at low abundance in the rhizosphere and appeared nearly absent from bulk soil, raising questions on the ecology and trophic mode of orchid mycorrhizal fungi beyond orchid tissues.

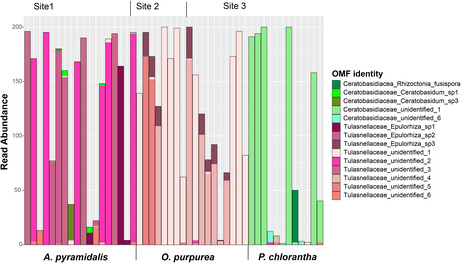

## 1 INTRODUCTION

Plant–microbe interactions have long been recognized for their importance for plant health and nutrient uptake and the last decade has brought an increasing awareness that microbes constitute a key component of plant ecology and evolution (Lyu et al., 2021; Trivedi et al., 2020; Vandenkoornhuyse et al., 2015), as part of the growing field of hologenomics (Alberdi et al., 2022; Lyu et al., 2021). Microbes have been shown to be involved in plant reproductive traits, local distribution and evolutionary patterns (Arifin et al., 2023; Helletsgruber et al., 2017; McCormick et al., 2018), calling for microbe interactions to be broadly considered when studying plant ecology, including habitat requirements and conservation of wild plant populations. Plant–fungal associations are ubiquitous and mycorrhizal associations occur in c. 90% of plant species (Brundrett & Tedersoo, 2018), and are generally characterized by transfer of nutrients, especially nitrogen (N) and phosphor (P), from fungus to plant, in exchange for carbohydrates (Smith & Read, 2008; van der Heijden et al., 2015). Orchid mycorrhiza is a unique type of endomycorrhiza that is distinguished by the development of specialized fungal structures called pelotons, which consist of coiled hyphae within the cells of orchid protocorms and roots (Rasmussen, 1995), as well as by transfer of carbon (C) from the fungus to the orchid (Gebauer et al., 2016; Gebauer & Meyer, 2003; Smith & Read, 2008). Orchid seeds are minute and devoid of endosperm, and the early stages of orchid growth are obligate dependent on infection with specific fungi. Protocorms rely on transfer of C from living hypha and on digestion of pelotons as their carbon source (Kuga et al., 2014; Smith & Read, 2008). All orchids, both terrestrial and epiphytic, are thus fully mycoheterotrophic in the early stages (Rasmussen, 1995). This lifestyle continues to a variable degree in the adult orchid, from fully mycoheterotrophic, achlorophyllous species, over partially mycoheterotrophic to autotrophic species, and most orchid species host orchid mycorrhizal fungi (OMF) in their roots (Gebauer et al., 2016; Girlanda et al., 2011; Herrera et al., 2022; Jacquemyn et al., 2017, 2021; McCormick et al., 2004, 2022; Zeng et al., 2021). Although it has been suggested that the fungal partner might benefit from the symbiosis by switching to decomposing orchid roots in later stages of infection, or from receiving C or ammonium from the orchid (Adamo et al., 2020; Cameron et al., 2008; Deiner et al., 2017; Fochi et al., 2017), it has not been broadly confirmed under natural conditions, and the relationship is assumed to be largely parasitic to the benefit of the orchid (Rasmussen et al., 2015; Rasmussen & Rasmussen, 2009). Some OMF have also been reported as saprotrophs (e.g. wood decomposers), mycoparasites, ectomycorrhizal fungi or as endophytes in non‐orchid plants (Põlme et al., 2020), but the ecology of most OMF detected from orchid tissues is poorly known (Dearnaley et al., 2012; Li, Yang, et al., 2021; McCormick et al., 2012; Põlme et al., 2020; Rasmussen & Rasmussen, 2014; Suetsugu et al., 2021). The most frequently detected OMF in terrestrial orchid species belong to the basidiomycete families Tulasnellaceae, Ceratobasidiaceae (both Cantherallales) and Serendipitaceae (Sebacinales) (Dearnaley et al., 2012; Rasmussen & Rasmussen, 2014). The specificity of the orchid–fungus relationship varies, but there is growing evidence that most of the orchid species have a limited range of fungi they associate with, and that this specificity affects their ecology (Li, Yang, et al., 2021; McCormick et al., 2018; Rasmussen et al., 2015). Orchid seeds depend on recruiting fungal partners from a narrow range of specific fungi in their environment in order to germinate, as there is no known vertical transfer of fungi to the seed (Rasmussen, 1995; Rasmussen et al., 2015). Correspondingly, germination and local distribution patterns of both terrestrial and epiphytic orchid species appear to depend on the distribution and abundance of OMF fungi in their substrate (Li et al., 2022; McCormick et al., 2016; Petrolli et al., 2022; Rock‐Blake et al., 2017). Different orchid species in the same habitat have been shown to each use distinct sets of OMF, hypothesized to be part of niche diversification (Cevallos et al., 2017; Jacquemyn et al., 2014, 2017; Oja et al., 2017; Petrolli et al., 2022). Use of and adaptation to different fungi allows for co‐existence in high‐diversity plant communities, and specialization to different fungi may be involved in orchid diversification and speciation processes as well (Arifin et al., 2023; Xing et al., 2017). A number of recent studies have documented a significant spatial turnover in fungal niches, especially for widespread orchid species (Duffy et al., 2019; Jacquemyn et al., 2016; Wang et al., 2019; Xing et al., 2020), while other species appear to have very narrow OMF preferences across their distribution range, particularly in the critical protocorm stage (Kaur et al., 2019; McCormick et al., 2021). How among‐population variation in OMF communities is involved in adaptation to site‐specific conditions have been less explored (Jacquemyn et al., 2018; Wang et al., 2023), and it also remains to be thoroughly explored how root‐associated OMF communities reflect spatial variation of available OMF fungi in the soil. Recent studies have shown that local abundance of orchid species, their population size and even resilience to climatic stress were correlated to distribution and abundance of their OMF in the soil (Janissen et al., 2021; McCormick et al., 2018). Knowledge on fungal preferences of threatened orchid species could provide promising perspectives in orchid conservation (Phillips et al., 2020). For instance, by screening the soil in potential habitats for abundance and distribution of specific OMF associated with a targeted terrestrial orchid species would reveal whether the habitat is promising for re‐introduction and/or assisted migration. Understanding of the spatial patterns of specific OMF strains in soil would allow a better recognition of factors determining local occurrence and abundance of terrestrial orchid species. While a few studies found that OMF strains were mainly identified in spots where their associated orchids were present (Li et al., 2022; McCormick et al., 2018), it is not generally known how common or rare soil OMF are and which trophic mode they exhibit. Knowledge on the broader ecology of OMF can help design management strategies for endangered orchid species by improving the growing conditions for the fungus involved (Whigham et al., 2021).

**FIGURE 1:**
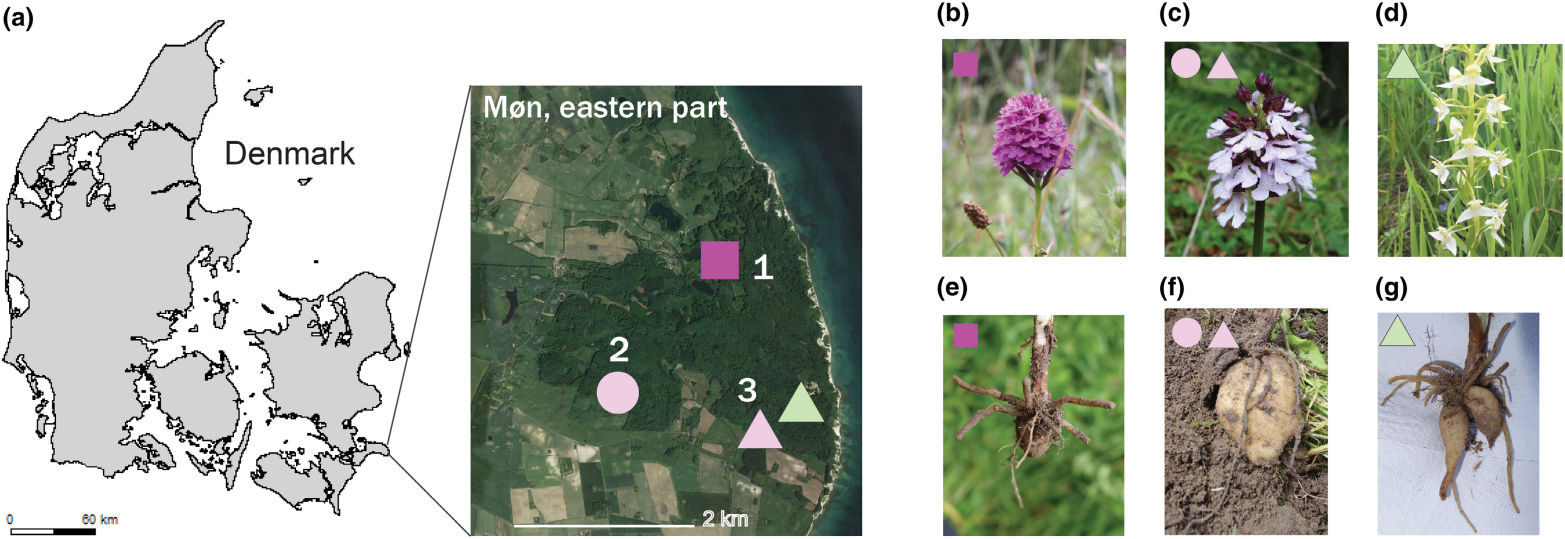
Sampling area and study species. (a) Location of the three study sites (1: Jydelejet, 2: Hoevblege and 3: Hundevaeng) on the isle of Møn in eastern Denmark, symbol colour indicates species sampled at each sites and symbol shape the location. (b) *Anacamptis pyramidalis*, the Pyramidal orchid, (c) *Orchis purpurea*, the Lady orchid, (d) *Platanthera chlorantha*, the Greater Butterfly orchid, (e) Root system of *A. pyramidalis*, (f) Root system of *O. purpurea*, (g) Root system of *P. chlorantha*. Root samples were collected from thin adventitious roots, and not from tubers.

In this study, we explored the spatial distribution in the soil of OMF partners of three co‐occurring terrestrial orchid species in a local orchid diversity hotspot in southeastern Denmark (Figure 1). Half of Denmark's 37 species of orchids are considered threatened, (Damgaard et al., 2020), and information on specific symbiotic associations as both pollinators and mycorrhizal partners are undescribed for most species (Damgaard et al., 2020). Myccorhizal partners in Scandinavian orchid populations are largely undocumented, and as OMF are known to vary within the distribution range of the host species (Duffy et al., 2019; Xing et al., 2020), a necessary first step was to identify specific OMF partners and whether co‐occurring species exhibit differential OMF preferences, as suggested from a growing number of studies (Li, Wu, et al., 2021). We selected three native orchid species that differ in rarity and abundance in Denmark, and assessed their root mycobiome with ITS metabarcoding at three localities, and compared soil mycobiome profiles for orchid rhizosphere soil and bulk soil at the localities.

First, we aimed to identify the myccorhizal partners of the selected three species in the northern part of their ranges, and test whether they associate with different orchid mycorrhizal partners when co‐occurring, following the pattern documented in similar studies. Then, we specifically aimed to test if the identified OMF are more abundant in the rhizosphere soil than in bulk soil, indicating a tight relation between occurrence of adult orchids and their OMF partners.

Addressing these objectives will enable us to understand better the habitat use and requirements of vulnerable Danish orchid species, and help guide conservation and management projects, such as identifying new localities suitable for re‐introduction and assisted migration.

## 2 MATERIALS AND METHODS

### 2.1 Study site and species

The eastern part of the isle of Møn in southeastern Denmark (Figure 1) is a local hotspot of orchid diversity, hosting 18 of the 37 orchid species occurring in Denmark (Pedersen & Faurholdt, 2010), all of which are terrestrial. The area is known for its calcareous soils providing species‐rich grassland and forest habitats protected within the Natura2000 network, and also forms a UNESCO Biosphere. As focal taxa, we chose three native orchid species that differ in rarity in Denmark, and which have overlapping distributions on Møn (Figure 1). The Pyramidal orchid *Anacamptis pyramidalis* (L.) Rich (Orchidoideae: Orchidiniae) has a very limited distribution in Denmark and is almost exclusively found in a single locality on Møn, and the species is listed as Vulnerable in Denmark (Moeslund et al., 2019). A few satellite populations of limited size have however recently been found in other parts in Denmark. The Lady orchid *Orchis purpurea* Huds. (Orchidoideae: Orchidiniae) is known from a handful of sites at Møn and also in western Denmark. The species has been assessed as Near Threatened in Denmark and Least Concern globally (Moeslund et al., 2019; Rankou, 2011). Both *A. pyramidalis* and *O. purpurea* occur in a broad belt from Southern and Western Europe, including the British Isles, and eastwards to Caucasus, and the populations in Denmark are at the northern margin of their distributions (GBIF, 2022; Pedersen & Faurholdt, 2010). The Greater butterfly orchid *Platanthera chlorantha* (Orchidoideae: Orchidiniae) is broadly distributed from Western Europe to Central and East Asia, and in Denmark the species is far more common than the other two, known from a number of regions in both eastern and western Denmark (Pedersen & Faurholdt, 2010). It can be locally abundant, with population size reaching thousands of individuals (DN, 2017). Nevertheless, it is listed as Near Threatened in Denmark (Moeslund et al., 2019).

### 2.2 Fieldwork

We collected root samples from the three focal species from three localities (Figure 1, Appendix A); Site 1 (Jydelejet): *A. pyradamidalis* (July 2017 + June 2018), Site 2 (Hoevblege): *O. purpurea* (June 2018) and Site 3 (Hundevaeng): *P. chlorantha* (July 2017 + June 2018) and *O. purpurea* (June 2018). All three sites are open calcareous grasslands with a high diversity of vascular plants (Hartvig et al., 2021) and are managed with cattle grazing to maintain an open landscape and prevent shrub encroachment. While Site 1 and 2 have a long history as protected natural grassland, Site 3 was cultivated farmland until 1995, and has only been protected since 2013.

We carefully collected one entire root from each individual to a 5‐mL tube and immediately placed it in a cooler with ice packages. For each root sample, we collected two soil samples: one sample collected from the soil surrounding the collected root, representative of rhizosphere soil, and another soil sample collected 1 m away from the orchid individual, in a random direction, and at the approximate same depth as the roots of the targeted plant (app. 5 cm), considered bulk soil. Each soil sample was collected with a sterile 50 mL Falcon tube, placed in a zip‐lock bag, which was then placed inside another zip‐lock bag (to avoid contamination) and stored in the cooler as well. The samples accumulated to 25 root samples and 49 soil samples for *A. pyramidalis*, 20 root samples and 40 soil samples for *O. purpurea* distributed on two sites, and 31 root samples and 62 soil samples for *P. chlorantha* (Appendix A).

### 2.3 Molecular work

We cleaned the orchid roots from visible soil in running water and then surface sterilized them with 30 s submersion in 1% NaOCl and three subsequent washes of 30 s each in sterile water. Root samples were then kept at −20°C until DNA extraction. All sterilization and DNA extraction procedures were conducted in a sterile laminar flow hood to minimize environmental DNA contamination. DNA from root samples were extracted with the DNeasy® PowerPlant Pro DNA isolation kit (Qiagen) following the manufacturer's instruction. First, samples were cut into small pieces, placed in liquid nitrogen and ground in sterile ceramic mortars to produce a coarse paste. Up to 50 mg of root tissue was used for each extraction column, which meant that the tissue for most samples were divided across two or three reactions. We only handled one root sample at a time in the flow hood for the grinding step, to minimize cross‐contamination. We used the manufacturer's instructions for the rest of the extraction protocol. DNA extracts stemming from the same root sample were then combined, if extraction had been conducted in multiple columns.

We extracted DNA from soil samples with DNAeasy® PowerSoil® Kit, following the manufacturer's instructions. We used 0.25 g of soil per sample, avoiding any visible root and tissue fragments. The DNA extracts were cleaned with DNAeasy® PowerClean® Pro Cleanup Kit (Qiagen), as applied in similar soil metabarcoding studies (Froslev et al., 2019).

Both root and soil samples were subjected to DNA metabarcoding targeting the entire ITS region, using the primers BITS (ACCTGCGGARGGATCA; Bokulich & Mills, 2013) and ITS4_KYO1 (TCCTCCGCTTWTTGWTWTGC; Toju et al., 2012) to facilitate a broad identification of fungal taxa in root and soils. Metabarcoding library preparation and Illumina MiSeq sequencing with V3 chemistry (2 × 300 bp) were conducted at the GSAF sequencing facility at Austin University, Texas, USA.

Some strains of basidiomycete OMF pose a challenge to amplify with standard ITS primers, due to very divergent ITS sequences (Moncalvo et al., 2006; Taylor & McCormick, 2008). We therefore supplemented the metabarcoding approach with direct PCR on root DNA samples using primers specifically for known OMF groups. To target Tulasnellaceae from root samples, we used the ITS5 forward primer (GGAAGTAAAAGTCGTAACA) in combination with modified ITS4 primers, the ITS4Tul (CCGCCAGATTCACACATTGA) and ITSTul2 (TTCTTTTCCTCCGCTGAWTA) (Taylor & McCormick, 2008), in separate reactions for each reverse primer. We used the DreamTaq PCR Master Mix (ThermoFisher Scientific, Lithuania) and applied the following PCR conditions: 3 min of initial denaturation at 95°C followed by 35 cycles of denaturation at 95°C for 30 s, annealing at 54°C for 30 s and elongation at 72°C for 45 s, and a final elongation step at 72°C for 5 min. To target Ceratobasidiaceae, we used the primer pair CeTh1 (TTCTCTTTCATCCACACACMCC) and CeTh4 (ACAGGATGCTCCAGGAATACC) (Porras‐Alfaro & Bayman, 2007), using an annealing temperature of 57°C, and otherwise the same master mix and cycling conditions as described for the Tulasnellaceae primers. PCR purification and Sanger sequencing were performed by Macrogen Europe (Amsterdam, The Netherlands), using the same primers as the in PCR reactions. The raw sequences were edited and assembled using Geneious 2019–2020 (Biomatters Inc., New Zealand).

### 2.4 Bioinformatic analyses of Illumina metabarcoding data

Adapter trimming, removal of low quality bases (*Q* < 20) and short reads (<75 bases) of raw Illumina reads was done using BBduk of the BBTools suite v 38.66 (https://jgi.doe.gov/data‐and‐tools/software‐tools/bbtools/). The raw data were imported to QIIME2 v 2021.11 (Bolyen et al., 2019) where primer and 5.8S sequences were stripped using Cutadapt (https://cutadapt.readthedocs.io/en/stable/) (Martin, 2011). OTUs were picked up at 99% similarity against UNITE database v 8.3 (released 2021‐05‐10) using VSEARCH (Rognes et al., 2016) and a close‐reference approach of QIIME2. The generated OTU table was exported to R for statistical analyses. Only forward reads containing the internal transcribed spacer 1 (ITS1) region of the eukaryotic ribosomal gene cluster were used in this study. All above‐mentioned bioinformatic procedures were performed in QIIME2 space unless otherwise stated.

### 2.5 Data analysis

#### 2.5.1 Mycobiome composition and diversity in orchid roots and soil

All analyses of metabarcoding datasets were conducted in R 4.2.0 with the packages phyloseq (McMurdie & Holmes, 2013) and vegan (Oksanen et al., 2020). First, we divided the dataset by sample type (root and soil), as both total read number and number of OTUs detected were much higher in soil than in root samples. Soil samples were rarified to 3000 reads/samples, and root samples to 200 reads/sample, as OTU accumulation above those values was minimal for most samples, and that setting allowed us to keep 48 of 78 root samples, and 133 of 155 soil samples. Initial exploration of the datasets showed a very limited effect of sampling year, and hence we grouped samples from different years in order to obtain higher sample sizes per host identity and site. We used Bray–Curtis dissimilarity index and pairwise permutational analyses of variance (PERMANOVA) with 999 permutations using the adonis function of vegan, to explore if composition of orchid mycobiomes differed with host identity and site. Similarly, we tested the effect of site, host identity and soil type (rhizosphere and bulk) on soil mycobiome, using the same settings. We identified OMF strains from the metabarcoding datasets as those belonging to either Tulasnellaceae or Ceratobasidiaceae (as only a single Serendipitaceae OTU with a few reads was detected from a single root sample) and compared their relative abundance in rhizosphere and bulk soil samples, for each host species, with Mann–Whitney‐Wilcoxon tests in R 4.2.0.

The Fungaltraits database (Põlme et al., 2020) was used to assess the assumed trophic modes of identified non‐OMF fungi.

#### 2.5.2 Diversity of OMF strains identified from orchid roots

For a more complete exploration of diversity of mycorrhizal fungi host in the three species, we combined the metabarcoding data with the results from Sanger sequencing with OMF specific primers. We extracted per sample presence–absence records of all OTUs identified as Tulasnellaceae and Ceratobasidiacea from the quality‐filtered, but none‐rarified metabarcoding dataset. To facilitate comparison between the two types of sequencing data at similar taxonomic levels, we clustered ITS sequences identified with Sanger sequencing at 99% sequence similarity using the de novo assembling function in Geneious Prime 2022 (Biomatters Inc., New Zealand), and added per‐sample occurrence of each resulting ITS cluster to the metabarcoding presence‐absence OTU table. Based on this combined dataset, we calculated number of OMF OTUs identified per samples and species, and proportion of shared and unique OTUs among the three species.

## 3 RESULTS

### 3.1 Orchid mycobiome identified with ITS metabarcoding

Metabarcoding analyses of rhizosphere and bulk soil samples yielded a median of 7839 reads per sample (150 samples, range 976–75,399 reads), while root samples had a median of 352 reads per sample (76 samples, range 3–45,369 reads), indicating lower level of input target fungal DNA in root samples. After rarefaction, the datasets included 48 root samples and 133 soil samples (Appendix A). Most discarded root samples were from *P. chlorantha* (19, Appendix A).

**FIGURE 2:**
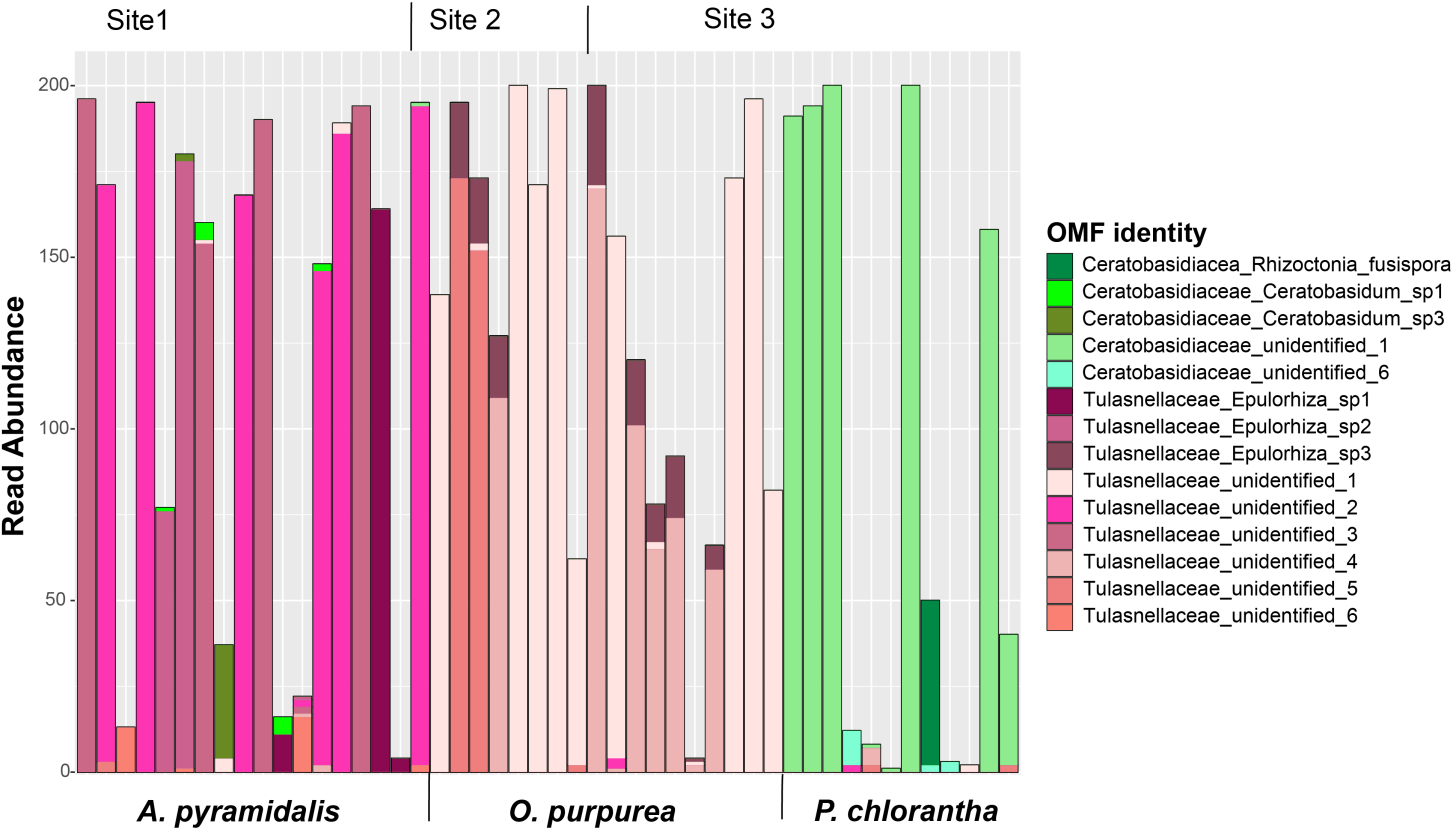
Read abundance of orchid mycorrhizal fungi (OMF) in root samples from *Anacamptis pyramidalis*, *Orchis purpurea* and *Platanthera chlorantha*, as identified with ITS metabarcoding. Each bar represents a single root sample. Data normalized to 200 reads per sample. For clarity, non‐OMF reads are not displayed.

The rarefied root dataset contained 144 fungal OTUs, with 80 OTUs found in *A. pyramidalis* roots, 58 in *O. purpurea* and 72 in *P. chlorantha*. The majority of reads from roots (60%) belonged to the two OMF families, Tulasnellaceae and Ceratobasidiaceae, with a lower proportion in *P. chlorantha* than in the other two species (Figure 2). The identity and abundance of OMF OTUs differed among the host species. *Anacamptis pyramidalis* and *O. purpurea* displayed a high abundance of differing OTUs of Tulasnellaceae, whereas *P. chlorantha* mainly hosted a single OTU of Ceratobasidiaceae. *Orchis purpurea* was associated with similar OMF OTUs across the two different collection sites (Figure 2). Remaining reads in the root samples were mainly unidentified at the order level (17%), or as Hypocreales (6%) and Eurotiales (5%) (both Ascomycetes). The most common non‐OMF OTUs identified to genus level was *Trichoderma* (mycoparasite, endophyte or saprophyte, Hypocreales), *Diaporthe* (plant pathogen, endophyte or litter saprophyte, Diaporthales), *Hannaella* (animal pathogen or saprophyte, Tremellales), *Ilyonectria* (plant pathogen and saprophyte, Hypocreales), *Exophiala* (animal pathogen or litter saprophyte, Chaetothyriales) and *Fusarium* (plant pathogen, endophyte or litter saprophyte, Hypocreales) (Põlme et al., 2020).

**FIGURE 3:**
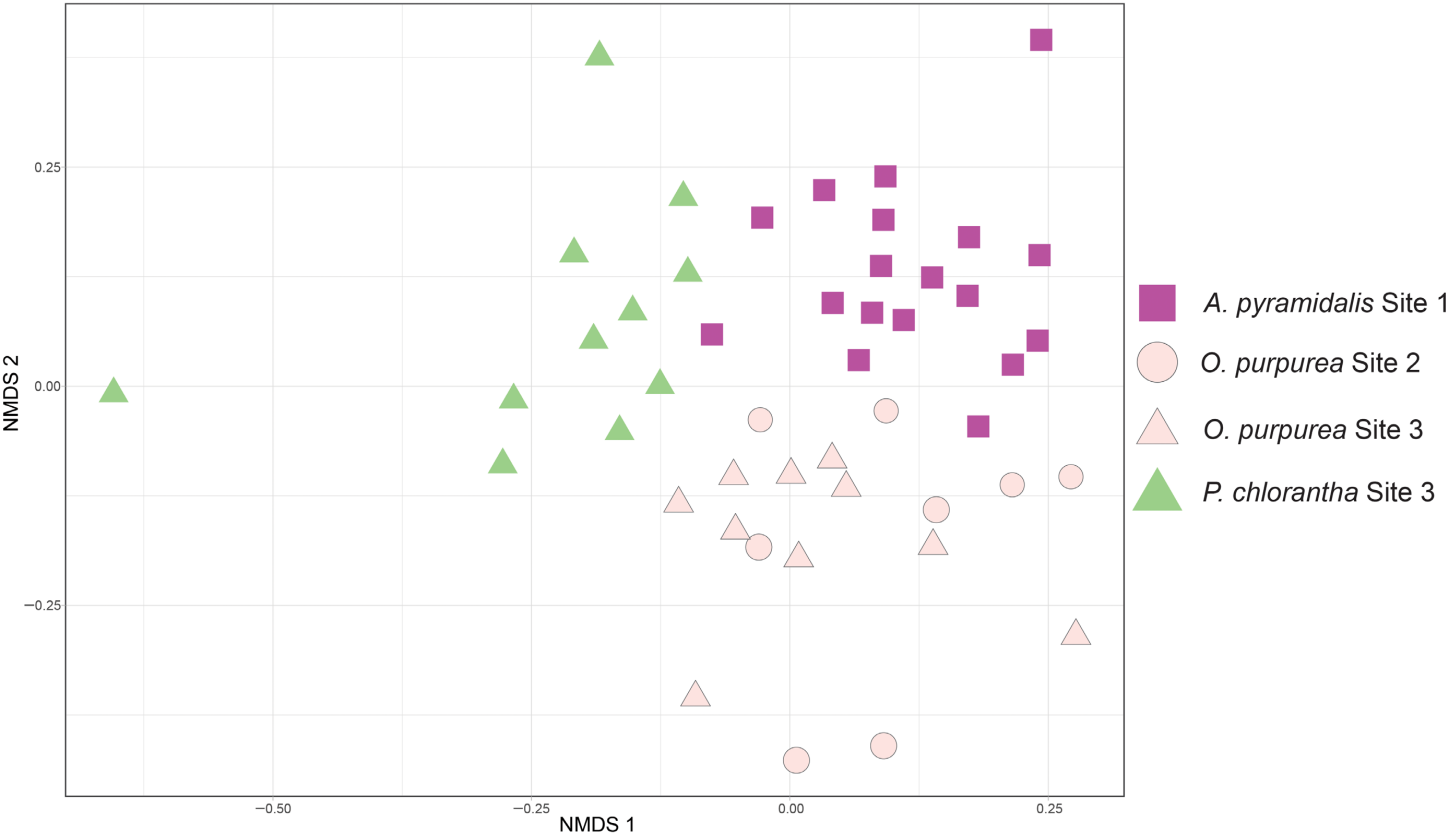
Beta diversity of orchid root mycobiomes, as identified with ITS metabarcoding. NMDS plot of root mycobiome composition in three orchid species (magenta, rose and green) collected at three sites (rectangle, circle and triangle).

The composition of the mycobiomes were significantly different among the three orchid species (Figure 3, permanova, *p* < .001), and only 21 of 144 OTUs were shared among all three species. There was no significant effect of site on mycobiome composition within *O. purpurea* (*p* = .13), as illustrated by similar root mycobiome profiles for roots of that species from Site 2 and Site 3 (Figure 3). There was a significant effect of host species within site, as mycobionts of *P. chlorantha* and *O. purpurea* differed at their site of co‐occurrence, Site 3 (Figures 2 and 3, *p* < .01). As 60% of the reads from root samples could be attributed to OMF OTUs, the difference in mycobiome among species is largely driven by difference in OMF associations, but the non‐OMF alone also differed significantly among the three species (permanova, *p* < .001).

### 3.2 Orchid mycorrhizal fungi identified in *A. pyramidalis*, *P. chlorantha* and *O. purpurea*

**FIGURE 4:**
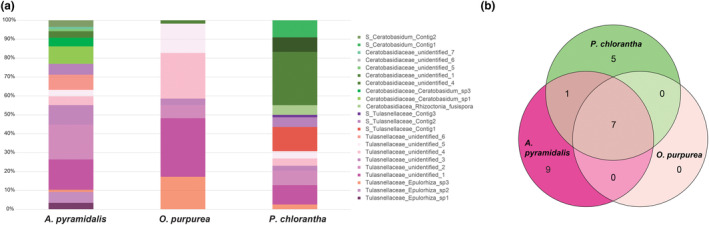
Orchid mycorrhizal fungi (OMF) community structure in three orchid species as identified with general fungal primers and OMF‐targeted sequencing. (a) Frequency of observed OMF OTUs in root samples of the three species. (b) Venn diagram showing the number of unique and shared OMF OTUs of the three species.

We detected 17 OTUs from the putative orchid mycorrhizal families Tulasnellaceae and Ceratobasidiaceae with the general fungal primers used in the metabarcoding approach. Sanger sequencing with specific Tulasnellaceae and Ceratobasidiaceae primers additionally identified three Tulasnellaceae OTUs and two Ceratobasidiaceae OTUs from *A. pyramidalis* and *P. chlorantha* roots, while the specific primers did not yield any PCR products from *O. purpurea* samples (Appendix B). Combining the data from both sequencing approaches, we identified 17 OTUs belonging to either Tulasnellaceae or Ceratobasidiaceae from *A. pyramidalis*, seven from *O. purpurea* and 13 from *P. chlorantha*. Most orchid roots associated with two to five different OMF strains, with a span from 0 to 7. On average, *A. pyramidalis* hosted 3.5 OTUs per individual root sample, *O. purpurea* 2.9 OTUs, while *P. chlorantha* had fewer OMF on average, 2.2. In two of the 31 analysed root samples from *P. chlorantha* we failed to detect any OMF, across the two methods. Seven Tulasnellaceae OTUs were detected in all three species, albeit in different frequencies (Figure 4). Nine OTUs (three Tulasnellaceae and six Ceratobasidiaceae) were specific to *A. pyramidalis*, and *P. chlorantha* hosted two Ceratobasidiaceae OTUs that were not found in the other species. In addition to species‐specific OTUs, the frequency of the shared OTUs, as well as the abundance for those identified with metabarcoding approach, diverged among the three species, resulting in different OMF profiles (Figures 2 and 4).

### 3.3 Soil mycobiome and abundance of orchid mycorrhizal fungi in soil

**FIGURE 5:**
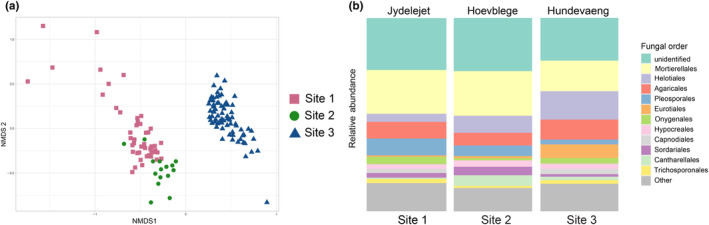
Soil mycobiome in three sites in eastern Denmark (a) NMDS plot of mycobiome composition in soil samples from the three study sites, (b) relative abundance of 12 most abundant fungal families identified from the soil.

After rarefaction, the metabarcoding approach recovered 688 fungal OTUs from the soil, of which less than half (312) were common for all samples, indicating high heterogeneity in soil mycobiome across the sampling area. Twenty‐four per cent of total reads were unidentified at order level, and the most abundant identified fungal orders were Mortierellales (19% of total reads), Helotiales (10%), Agaricales (9%) and Pleosporales (5%) (Figure 5). The most abundant OTUs identified to genus level was *Morteriella* (soil saprotroph and known to be root‐associated), *Solicoccozyma* (soil saprotroph and endophyte) and *Hygrocybe* (soil symbiotroph and unspecified endosymbiotroph) (Põlme et al., 2020). The three sampled sites contained significantly different soil mycobiomes (permanova, *p* < .001), with Site 3 distinct from Site 1 and Site 2 soil, which had some overlap (Figure 5). The differences among the sites were apparent in relative abundance of fungal orders, revealing a higher relative abundance of Mortirellales at Site 1 and Site 2, and a higher proportion of Helotiales and Agaricales at Site 3. Site 2 had a larger share of Cantherallales, representative of Tulasnellaceae and Ceratobasidiaceae, compared to the other sites.

**FIGURE 6:**
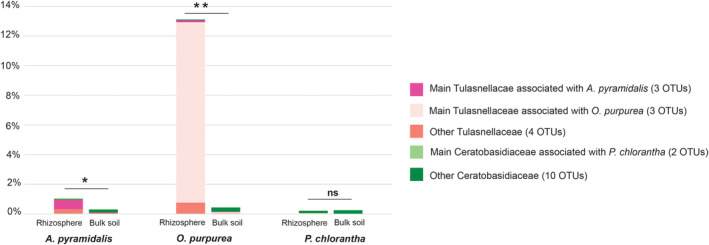
Abundance of orchid mycorrhizal fungi (OMF) detected from ITS metabarcoding of rhizosphere and bulk soil, respectively, per orchid species. OMF colour coded according to their main association (see Figure 2). *p < .001, **p < .0001, ns, not significant.

The overall mycobiome composition did not differ significantly between rhizosphere and bulk soil samples within each site, nor between rhizosphere and bulk soil samples from the same species (permanova, *p* = .55 for soil type within site, *p* = .4 for soil type within species). In their abundance of OMF, however, the rhizosphere and bulk soils did differ. We detected 22 OTUs belonging to the OMF families, Tulasnellaceae and Ceratobasidiaceae, from the soil, which together constituted 1.8% of the total reads across soil samples. The two most common OMF OTUs identified for *O. purpurea* (Tulasnellaceae_unidentified 1 and Tulasnellaceae_unidentified _4, Figure 2), made up 72% of the total OMF reads from soil samples, while the rest of the OMF OTUs were detected at very low abundance (Figure 6). The abundance of OMF reads in rhizosphere soil samples were 10‐fold higher than in bulk soil samples, 3.3% vs. 0.3%, which was mainly driven by a few *O. purpurea* rhizosphere samples with high OMF read abundance (*p* < .001, Mann–Whitney–Wilcoxon test between rhizosphere and bulk samples for *O. purpurea*). There was also a significantly higher proportion of OMF reads in rhizosphere than bulk soil for samples associated with *A. pyramidalis* (*p* < .01), while no difference in OMF read abundance between rhizosphere and bulk soil for *P. chlorantha* (*p* = .9, Figure 6). In terms of occurrence, 91% of the rhizosphere samples contained OMF strains, while this was only true for 69% of the bulk soil samples.

## 4 DISCUSSION

This study represents the first detailed record of OMF associations in populations of native Danish orchid species, and improves our understanding of the fungal requirements of these rare and protected species. Each of the orchid species studied was associated with a distinct set of specific fungi, both for OMF and for non‐OMF fungi, supporting the theory that partnerships with specific microorganisms constitute part of a species' ecological niche. A similar selection of orchid mycorrhizal partners in *Orchis purpurea* across two sites with differing soil mycobiomes indicates a strong host effect on plant mycobiome, in line with the hologenomics theory (Alberdi et al., 2022; Rosenberg & Zilber‐Rosenberg, 2018). The very low abundance of OMF in the soil may point to highly specific fungal requirements of orchids, but also highlights our limited understanding of the broader ecology of OMF and how it depends on the physical and chemical properties of the soil.

### 4.1 Differential use of mycorrhizal fungi in an orchid hotspot in Denmark

The differential profiles of OMF in three Danish orchid species agrees with a growing body of literature demonstrating high degree of species‐specific OMF associations in both terrestrial and epiphytic orchid species (Arifin et al., 2022; Girlanda et al., 2011; Jacquemyn et al., 2014; Kinoshita et al., 2016; McCormick et al., 2016; Petrolli et al., 2022). We identified a relatively high number of OMF partners for each orchid species and a considerable overlap in fungal taxa (Figure 4). However, the difference in abundance among OMF strains revealed by the metabarcoding approach suggests that each species is mainly associated with a few OMF OTUs, with the rest found in very low abundance, as reported for a range of terrestrial orchids (Mennicken et al., 2023). This pattern matches the apparent generalism concept suggested by Shefferson et al. (2019), in which a species would be specialized towards a few fungi providing unique resources, across habitats, but also associated with other fungi that contribute with functionally redundant resources. As such, the few specific fungi for an orchid could be considered part of its core microbiome (Neu et al., 2021). The non‐mycorrhizal root endophytes identified represented both orders and taxa previously reported as orchid endophytes (e.g. *Fusarium*, *Trichoderma*) (Ma et al., 2015), as well as others not documented from orchids before, but generally known as soil fungi and/or endophytes/pathogens. Our study showed that these non‐OMF fungi differed among species, indicating that infection by non‐mycorrhizal endophytes are also influenced by the host and probably has functional significance for the orchid. This finding supports the notion that the microbiome as a whole, including both mycorrhizal and non‐mycorrhizal fungi, is relevant and deserves further exploration in understanding the interactions and dynamics within orchid‐microbe associations. It is also worth considering that this sampling represents specificity only in the adult life stage, and that earlier stages might be even more specific in fungal requirements than observed here (Lespiaucq et al., 2021; Rasmussen et al., 2015).

*Orchis purpurea* appears more specialized in its OMF associations than the other two species, with the lowest number of OMF OTUs detected, and a similar OMF profile across two localities with different soil mycobiomes (Figure 5). At the sites on Møn, *Orchis purpurea* seems confined to Tulasnellaceae, whereas studies from populations in Italy, France and Belgium found it to associate with both Tulasnellaceae and Ceratobasidiacea, and also to receive both N and C from its fungi (Girlanda et al., 2011; Lievens et al., 2010). Highly similar fungal strains (>99% sequences similarity) to the specific OTUs we detected in *O. purpurea* have been detected from *O. purpurea* at other sites in Europe (Appendix B), indicating consistent preferences for similar fungi across its distribution area. The interaction between habitat variation and OMF associations for orchids remains to be thoroughly explored, but a number of studies point to a considerable effect of habitat on detected OMF community species (Duffy et al., 2019; Jacquemyn et al., 2016; Wang et al., 2023; Xing et al., 2020). Differences in sampling efforts can thus greatly effect conclusions drawn on specificity, as the degree of specificity is influenced by environmental factors (Kaur et al., 2021; Shefferson et al., 2019), and OMF profiles based on a few populations and/or limited proportion of the total distribution area for a species are not necessarily representative of species‐wide OMF associations.

*Anacamptis pyramidalis* and *P. chlorantha* both associated with higher numbers of OMF OTUs than *O. purpurea*, pointing towards adaptation to a slightly broader but still restricted range of fungi. Our record of fungal use in *A. pyramidalis* is the first reported in Scandinavia and Northern Europe, and the results match the only other record of the species from central‐eastern Europe, where it was interpreted to be a specialist associating with a few strains of both Tulasnellaceae and Ceratobasidiaceae origin (Těšitelová et al., 2022; Vogt‐Schilb et al., 2020).

With its lower average number of OTUs per individual, and a higher turnover rate of fungal partners, *P. chlorantha* may be considered more generalist in its fungal associations than *O. purpurea* and *A. pyradamidalis*, as well as less dependent on fungi in the adult stage, since we failed to identify OMF from several root samples of this species. The differing levels of specificity among the three species might be associated with their distribution and rarity in Denmark. *Anacamptis pyramidalis* and *O. purpurea* both have very restricted distribution areas in Denmark, while the presumably less specific *P. chlorantha* can be common in some regions, and with very large population sizes (DN, 2017). Although rarity and distribution range in general do not seem to be correlated with fungal specificity in orchid species, smaller‐scale distribution and population size might be (Bailarote et al., 2012; Calevo et al., 2020; Kaur et al., 2019).

Different European and North American species of *Platanthera* have been described to favour Ceratobasidiaceae (Esposito et al., 2016; Jacquemyn et al., 2021; Kaur et al., 2019). Oja et al. (2017) also found *P. chlorantha* to be associated with Ceratobasidiaceae, and to a lesser extent with Tulasnellaceae, and a similar average number of OMF OTUs per root, 3.3. Due to difference in methodology and primer bias, comparisons among different studies targeting the same species can be challenging, however. The difference between the OMF profiles for *P. chlorantha* detected with metabarcoding only and with data combined from metabarcoding and targeted primers (Figures 3 and 4) highlights the critical issue of primer bias. An approach with general primers identify a broad diversity of root endophytes, including some strains from OMF families, but fail to detect certain specialized fungi especially from Tulasnellaceae, as also acknowledged in other studies (Kaur et al., 2019; Taylor & McCormick, 2008; Vogt‐Schilb et al., 2020). These specific Tulasnellaceae may be generally under‐detected due to primer mismatch with general primers and thus underrepresented in reference databases, hindering a broader assessment of their abundance and distribution also outside orchid tissues. Other studies of *Platanthera* might have failed to identify specific Tulasnellaceae strains due to primer mismatch, and Tulasnellaceae do seem to constitute a significant part of the OMF community of the closely related *P. bifolia* (Vogt‐Schilb et al., 2020), as seems to be the case here for *P. chlorantha*.

To avoid primer bias and overcome reference database shortcomings, PCR‐free methods such as metagenomics appear promising (Azevedo‐Silva et al., 2021; Hernández‐Álvarez et al., 2022; Kaushal et al., 2020). In combination with whole genome sequencing of OMF, this would improve both references database and detection methods, and may yield significantly different insights for species level specificity as well as a broader understanding of occurrence and distribution of OMF outside orchid tissue.

### 4.2 Orchid mycorrhizal fungi occur at low abundance in the soil

The orchid root samples were dominated by OTUs from known OMF families, but these fungi were only found in very low abundance in the soil, affirming an active process of acquisition of specific fungi by the orchid from the soil. The abundance of specific OMF fungi was higher in rhizosphere soil than in bulk soil, indicating either that orchids occur only where the appropriate fungi occur in adequate abundance, as reported in McCormick et al. (2016), (but see Janissen et al. (2021)), and/or that the orchid has a positive effect on the abundance of the fungus in the rhizosphere soil. Low abundance of specific OMF in the soil has also been reported for other terrestrial species, such as *Platanthera preclaera* (Kaur et al., 2019), *Diuris* (Egidi et al., 2018), *Orchis*, *Neottia*, (Oja et al., 2015), *Anacampti*s, *Ophrys* (Voyron et al., 2017) and appears to be a general constraint on orchid germination and population growth (Jiang et al., 2022; Kaur et al., 2021; McCormick et al., 2016). Soil distribution and abundance of OMF are likely underestimated due to primer bias of general metabarcoding primers, and in this study might contribute to the lack of difference between rhizosphere and bulk soil OMF abundance for *P. chlorantha*.

The low likelihood of seeds reaching a patch of suitable habitat for germination is mitigated by the reproduction strategy of orchids, dispersing thousands of tiny seeds per capsule, and thus maximizing the change of landing in a spot with sufficient abundance of the right OMF. The very low abundance of OMF in soil however also leaves unanswered questions about the ecology of OMF when they are not associated with orchids. The ecology of some OMF is well known, for example, ectomycorrhizal lineages as Russulaceae (Rock‐Blake et al., 2017) or wood decomposers, such as the preferred OMF of *Tipularia discolor* (McCormick et al., 2021). Many Tulasnellaceae and Ceratobasidiaceae OMF are assumed to be general saprotrophs or wood decomposers (Dearnaley et al., 2012; Kohler et al., 2015; Rasmussen et al., 2015), but the very low abundance of OMF in soil reported does not seem to align well with this assumption. The work of Calevo et al. (2021) suggests that older orchid roots and other adult individuals are important reservoirs for OMF for recruitment by seeds and new roots, and that the soil represent less of a resource for fungal recruitment. Selosse (2014) also suggested that orchid roots present a refuge for OMF fungi. This would explain the patchy occurrence and increased germination rate in vicinity of adult orchids (Jacquemyn et al., 2012; McCormick et al., 2016), but would also make it even more difficult for orchids to establish new populations. Another possibility is that OMF are persistent in a network as endophytes in neighbouring plants, as they have been reported from a number of other species (reviewed in Selosse et al., 2021). A Ceratobasidiaceae OTU detected in this study was very similar to root endophyte OTUs detected from several crop species in Europe and USA (Appendix B). It is likely that Tulasnellaceae have been fundamentally overlooked in endophyte community studies in herbs, as they evade detection with the standard fungal barcoding primers (Kaur et al., 2021; Taylor & McCormick, 2008; Vogt‐Schilb et al., 2020). Investigation of endophyte networks in plant communities with OMF targeting primers or with metagenomics approaches would help elucidate the broad occurrence of OMF, and whether non‐orchid neighbouring plants could act as reservoirs for OMF (Selosse et al., 2021; Selosse & Martos, 2014). Many fungi exhibit flexibility in their carbon acquisition strategies (Harder et al., 2023; Selosse et al., 2018), and OMF fungi are known to possess saprotrophic abilities (Dearnaley et al., 2012). OMF fungi could thus function as root decomposers for non‐orchids hosts, and may also digest roots that are shed by terrestrial orchids after fruiting, as similarly suggested by Adamo et al. (2020).

### 4.3 Implementation of knowledge on OMF profiles in conservation strategies for orchid species

The very low abundance or even absence of the identified species‐specific OMF in the soil samples demonstrated here as well as in other recent studies (Egidi et al., 2018; Kaur et al., 2019; Oja et al., 2015; Vogt‐Schilb et al., 2020) limits the potential of screening new localities for their fungal communities and apparent ability to support new populations of orchids. However, identification of fungal partners can form an important part of conservation initiatives as habitat restoration and re‐introduction projects. Especially ecology and trophic modes of OMF would be beneficial for more targeted habitat management. Whigham et al. (2021) found that the presence of a threatened forest‐dwelling orchid was strongly correlated with soil concentration of its main OMF partner, which again was associated with availability of dead wood available for decomposing. Management that increases the amount of dead wood would thus be suggested to benefit the orchid through a positive effect on its fungal partner (Whigham et al., 2021). If screening of root endophytes across plant communities at orchid localities are able to identify OMF in non‐orchid plants this could potentially be an alternative method to identify suitable habitats for target orchid species, and/or provide an incentive to management practices that benefit other potential non‐orchid OMF hosts.

Recent field studies (Jiang et al., 2022; Těšitelová et al., 2022) have also demonstrated that the addition of mycorrhizal fungal cultures isolated from roots and protocorms of target orchid species, increased their germination rates in restored grasslands, showing a promising approach to management of threatened orchids. An important factor to consider in inoculation‐assisted restoration or habitat management is the potentially differing OMF needs from adult orchids, from which most of our knowledge on OMF association stem, to seeds, protocorms and early seedlings (Rasmussen et al., 2015). We attempted to identify the fungi involved in germination for *A. pyramidalis*, *O. purpurea* and *P. chlorantha* with the seed packet approach (Rasmussen & Whigham, 1993), burying seeds packets from all three species across the three localities. Unfortunately very low germination rates, likely associated with the extensive drought across Europe in the summer of 2018, limited the usability of the experiment (of 300 seed packages left at the localities, only seven contained protocorms after 19 months). The few fungi we managed to identify originated from *A. pyramidalis* protocorms, and were all recognized as Tulasnellaceae, suggesting that OMF associations in protocorms of this species are more narrow than in adults. This would put *A. pyramidalis* in a ‘nested with gain’ temporal turnover category (Lespiaucq et al., 2021; Rasmussen et al., 2015), but the experiments would need to be repeated to confirm the pattern. Interestingly, we observed germination of *A. pyramidalis* at Site 3, where it is currently not present, and where we did not detect its main OMF partners. This highlights the patchy occurrence of OMF fungi and the seemingly stochastic variation in germination success of orchids.

### 4.4 Conclusion

The highly specific fungal associations in orchids can be viewed as a model of the importance of microbionts for plant ecology and distribution. The differential OMF profiles in three co‐occurring orchid species in this study emphasizes the host‐dependent effect on plant microbiomes as predicted in the hologenomics theory, and the role of host–microbiome interactions in shaping ecological niches in co‐occurring plant species. Due to orchids' obligate dependence on fungal symbioses for germination, conservation plans for threatened orchids would benefit from including aspects of their fungal partners. A better understanding of the ecology and trophic modes of OMF, and whether they, for example, are dependent on specific environmental resources or they double as endophytes in neighbouring plants, is needed for a proper inclusion of orchid mycorrhizal partners in practical habitat management strategies.

## AUTHOR CONTRIBUTIONS

**Ida Hartvig:** Conceptualization (equal); data curation (supporting); formal analysis (lead); funding acquisition (equal); investigation (lead); methodology (lead); project administration (lead); visualization (lead); writing – original draft (lead); writing – review and editing (lead). **Chatchai Kosawang:** Conceptualization (equal); data curation (lead); formal analysis (supporting); methodology (supporting); writing – review and editing (supporting). **Hanne Rasmussen:** Conceptualization (equal); methodology (supporting); writing – original draft (supporting); writing – review and editing (supporting). **Erik Dahl Kjær:** Conceptualization (equal); funding acquisition (equal); writing – original draft (supporting); writing – review and editing (supporting). **Lene Rostgaard Nielsen:** Conceptualization (equal); funding acquisition (equal); investigation (supporting); methodology (supporting); project administration (supporting); writing – original draft (supporting); writing – review and editing (supporting).

## CONFLICT OF INTEREST STATEMENT

The authors declare no competing interests.

## Data Availability

ITS sequences from barcoding of root samples are available at NCBI Genbank, accession numbers OP537795–OP537812, OP502032–OP502041. Raw Illumina NGS data are available at University of Copenhagen's Electronic Research Data Archive (ERDA) at https://doi.org/10.17894/ucph.4663e425‐0266‐490c‐8962‐4bc26c825206.

## APPENDIX A

### A.1

Number of root samples and associated soil samples collected from three orchid species at localities at Møn, Denmark, as well as number of samples retained after rarefaction of metabarcoding datasets.

**Table:**

Site	Year	Species	Number of root samples	Number of root samples after rarefaction	Number of soil samples	Number of soil samples after rarefaction
3. Hundevaeng	2017	*P. chlorantha*	11	5	22	22
	2018	*P. chlorantha*	20	7	40	31
	2018	*O. purpurea*	10	10	20	18
2. Hoevblege	2018	*O. purpurea*	10	8	20	15
1. Jydelejet	2017	*A. pyramidalis*	10	7	19	19
	2018	*A. pyramidalis*	15	11	30	28
Total			76	48	151	133

## APPENDIX B

### B.1

Identified orchid mycorrhizal fungi (OMF) from roots of *Anacamptis pyramidalis*, *Orchis purpurea* and *Platanthera chlorantha* and their nearest NCBI genbank matches. Occurrence of all identified Tulasnellaceae and Ceratobasidiaceae fungi identified with ITS metabarcoding and Sanger sequencing, and the nearest match on NCBI genbank of their representative sequences. OMF ID starting with “S_” indicates identification with OMF‐specific primers and Sanger sequencing; all others were identified with metabarcoding using broad primers.

**Table:**

OMF ID	*A. Pyramidalis*	*O. Purpurea*	*P. Chlorantha*	Nearest match NCBI Genbank to UNITE representative sequence	Source nearest match
Tulasnellaceae_unidentified_1	x	x	x	99.46% KJ789936/GQ907256	*Anacamptis morio* root (Italy) and *Orchis anthropophora* root (Belgium)
Tulasnellaceae_unidentified_2	x	x	x	98.35% DQ925526	*Cyprepedium reginae* root (WV, USA)
Tulasnellaceae_unidentified_3	x	x	x	100% GQ907264	*Orchid militaris* roots (Belgium)
Tulasnellaceae_unidentified_4	x	x	x	100% JF926492	*Orchis purpurea* roots (Italy)
Ceratobasidiaceae_unidentified_1	x	x	x	100% OL685280	*Platanthera bifolia* protocorm (Czech Republic)
Tulasnellaceae_unidentified_5	x	x	x	100% GQ907279	*Orchis purpurea* roots (Belgium)
Tulasnellaceae_Epulorhiza_sp1	x			98.76% EU218890/98.41% KP868543	*Epulorhiza* sp. UAMH 5430/*Ophrys bertolonii* roots (Italy)
Tulasnellaceae_Epulorhiza_sp2	x			98.65% EU418851/98.64% GQ907274	*Serapias strictiflora* root (no locality information), *Orchis militaris* root (Belgium)
Tulasnellaceae_unidentified_6	x			99.44% KJ188456/GQ907271	*Neottia ovata* root (Czech Republic)/*Orchis militaris* root (Belgium)
Ceratobasidiaceae_unidentified_4	x			99.73% MH855687/MH854971	*Rhizoctonia fraxini* (no locality information)/ *Moliniopsis klebahnii* (no locality information)
Ceratobasidiaceae_Ceratobasidum_sp1	x			100% KY271870	*Neotinea ustulata* roots (Austria)
Tulasnellaceae_Epulorhiza_sp3	x	x	x	100% DQ925553	*Cypripedium parviflorum* root (KY, USA)
Ceratobasidiacea_Rhizoctonia_fusispora			x	99.42% HQ441575	*Rhizoctonia fusispora* (no locality information)
Ceratobasidiaceae_unidentified_5	x			98.64% HG936897	*Zea* field bulk soil (Germany)
Ceratobasidiaceae_unidentified_6			x	100% MT775823	*Spiranthes spiralis* (no locality information)
Ceratobasidiaceae_unidentified_7	x			99.78% MH861744/OM045908/MZ395973/MW999187	*Rhizoctonia solani*/*Phaseolus vulgaris* (IH, USA)/*Triticum aestivum* (USA)/ *Daucus carota* (Sweden)
Ceratobasidiaceae_Ceratobasidum_sp3	x			99.60% KC243943	*Gymnadenia conopsea* root (Czech Republic)
S_Tulasnellacae_Contig1			x	98.53% JX649078	Orchid root (Belgium)
S_Tulasnellacae_Contig2	x		x	99.57% JX649082	Orchid root (Belgium)
S_Tulasnellacae_Contig3			x	99.83% KJ188454	*Neottia ovata* protocorm (Czech Republic)
S_Ceratobasidiaceae_Contig1			x	100% OL685280	*Platanthera bifolia* protocorm (Czech Republic)
S_Ceratobasidiaceae_Contig2	x			98.1% JN683860	*Orchis tridentata* root (no locality information)
